# Soundscapes and Selection: Morphology, Ecology, and Evolutionary Dynamics of Weberian Apparatus in the Ictalurid Catfish

**DOI:** 10.1093/iob/obag061

**Published:** 2026-10-19

**Authors:** J C Hoeflich, J Liu

**Affiliations:** Department of Integrative Biology, University of California, Berkeley, CA 94720, USA; Museum of Paleontology, University of California, Berkeley, CA 94720, USA; Museum of Vertebrate Zoology, University of California, Berkeley, CA 94720, USA; Department of Integrative Biology, University of California, Berkeley, CA 94720, USA; Museum of Paleontology, University of California, Berkeley, CA 94720, USA

## Abstract

The Weberian apparatus is an innovation serving as the primary sound-conduction path for otophysan fishes. In this study, we investigate how this system of North American catfishes (Ictaluridae) varies across diverse ecological landscapes. By leveraging CT scanning and geometric morphometrics on 136 specimens across 47 species, we mapped the evolutionary trajectories of three critical components: the scaphium, tripus, and peripheral structures, including the associated vertebrae and ligament insertions. Our findings challenge conventional expectations that the apparatus is phylogenetically conserved. While phylogenetic signal is significant but low, we discovered exceptionally high morphological disparity driven by ecological selective forces. Factors such as stream velocity, climate, and even body coloration correlate strongly with ossicle shape, reflecting functional adaptations to specific micro-habitats. Crucially, we identified a clear signal of modularity; the scaphium and tripus demonstrate decoupled evolutionary rates, allowing them to evolve independently. This modularity suggests that the Weberian apparatus is not a rigid unit, but a highly plastic system that ictalurids might be used to fine-tune their sensory systems to adapt to ecological pressures.

## Introduction

The Weberian apparatus of otophysan fishes, the clade containing carp, catfish, tetras, and South American knifefishes, is arguably the most common multiple ossicular ear in vertebrates. Despite being less well-known than the mammalian middle ear, the Weberian apparatus is found in over 10,000 extant species of freshwater fishes (Nelson et al. 2016), almost double the 6600 species possessing the mammalian middle ear (Burgin et al. 2018). It has been claimed to play a role in the overwhelming evolutionary success of the otophysan fishes (Chardon and Vandewalle 1996). The Weberian apparatus is a sound conduction adaptation similar in function to the mammalian middle ear, transmitting sound from the anterior chamber of the gas bladder along a series of up to four ossicles (tripus, intercalarium, scaphium, and claustrum) connected by ligaments to the sinus impar and inner ear. The apparatus develops from the first four or five vertebra, with varying numbers fusing with the parapophyses into the parapophysitic sheet that may partially or fully enclose the anterior chamber of the gas bladder in catfish (Weber 1819; Alexander 1962; Alexander 1964; Chardon and Vandewalle 1996). The overall apparatus has been shown to be essential in increasing the hearing frequency range and enhance hearing sensitivity for otophysan fishes (Poggendorf 1952). While it is known that variation exists in the form and function of the Weberian apparatus (Bird and Hernandez 2007; Boyle and Herrel 2018; Mélotte et al. 2018), the evolutionary patterns and drivers of this variation is still unclear.

The catfishes, order Siluriformes, are known to have high morphological variation in the Weberian apparatus (Chardon and Vandewalle 1996). They exhibit both meristic and morphometric variations such as the number and the shapes of the ossicles. It has also been suggested that acoustic sensitivity of catfish varies with degree of gas bladder encapsulation created by the peripheral osteological structure of the Weberian apparatus (Lechner and Ladich 2008). Morphological studies in other otophysan fishes, particularly Cypriniformes (Bird and Hernandez 2007) and Characiformes (Boyle and Herrel 2018), have implied associations between form and ecology such as stream velocity. However, this question is complicated by the issue of phylogeny in catfish. While the catfishes of Africa, Asia, and South America are exceptionally diverse, the co-occurrence of families and the uncertain phylogenetic relationships (Teugels 1996; Kappas et al. 2016; Nelson et al. 2016) are major complicating factors to deduce the ecological impact on morphology.

The Ictaluridae, the North American freshwater catfishes, present an ideal system to investigate the relationship of morphology and ecology under phylogenetic framework as well as evolutionary dynamics through time of the apparatus. Ictalurids are the only native catfish throughout most of their range, have mostly resolved phylogenetic relationships (Arce-H et al. 2017; Janzen et al. 2023), and, unlike the plotosids of Australia or *Silurus* in Europe, which are also the only catfish clade throughout most of their range, they are both speciose and ecologically diversified (Nelson et al. 2016; Burr and Warren Melvin 2020). Furthermore, our previous work in the ictalurid genus *Noturus* has revealed considerable variation in the morphology of the Weberian apparatus, as well as significant correlations with some environmental factors (Hoeflich and Liu 2025). The purpose of this study is to investigate evolutionary patterns in the morphology of the Weberian ossicles, particularly the scaphium and tripus, and Weberian bony structures peripheral to the ossicles, in ictalurid catfishes. We test the influences of ecology, phylogeny, and modularity as well as the rates of morphological change and potential explanatory evolutionary models.

## Materials and methods

### Specimens, CT scan, and segmentation

We sampled 47 out of the 52 valid Recent species of Ictaluridae (Fricke et al. 2026) for this study. Two species, *Satan eurystomus* and *Trogloglanis pattersoni*, were excluded because of documented reduction of the Weberian apparatus to highly reduced, possibly non-functional ossicles (Lundberg 1982; Lundberg et al. 2017). We excluded two others, because they are under active taxonomic revisions: *Ictalurus ochotenarai*, which may be nested within the monophyletic *I. dugesii* [22]; and *Prietella lundbergi*, whose specimens are being reassessed. A population of *I. pricei* is revised into a new species, *Ictalurus nazas* (Avila-Treviño et al. 2025), published following the completion of our data collection and analysis. We are not certain whether the *I. pricei* specimens in this study belong to *I. pricei* or *I. nazas*. Therefore, we re-ran our analyses without the *I. pricei* specimens and included these results in the supplementary materials. From each of the remaining species except *Noturus stanauli*, we sampled at least two specimens. In cases where more than three fish were preserved in the same jar, we scanned all specimens but only used the largest three.

A total of 168 specimens were scanned and, after selecting the largest specimens and excluding likely juvenile individuals for each species, 137 specimens were included in this study. The specimens were loaned from a broad range of institutions, with 30 coming from the California Academy of Sciences, 27 from the University of Kansas, 26 from Tulane University, 23 from the Illinois Natural History Survey, 10 from the Academy of Natural Sciences of Drexel University (ANSP), nine from the North Carolina Museum of Natural Sciences, nine from the Field Museum of Natural History, and three from The Ohio State University at Marion (OSUM).

All specimens, except those from the OSUM, were scanned by JH at the Functional Anatomy and Vertebrate Evolution lab at University of California, Berkeley, using a Phoenix Nanotom 180m. The specimens were scanned with the Weberian apparatus as the region of interest, with voxel sizes ranging from 5.33 to 33.3 µm, current 70 to 130µA, and voltages 80 to 120 kV. The three OSUM specimens belong to the recently extinct species *Noturus trautmani*, were instead scanned at the Center for Electron Microscopy and Analysis facility in Ohio State University by Nate Shoobs (80–95 kV, 100 µA, and 8.7–10.8 µm per voxel) using a Thermo Scientific HeliScan microCT. All scans are uploaded to MorphoSource (project ID 000656686). Specific scan parameters for individual specimens are included in the Specimen Data Supplement.

Specimens were segmented in Avizo (Thermo Scientific 2021), primarily using the gray-value threshold, paintbrush, and lasso tools. Degrees of ossification, particularly of the claustrum, was variable. In order to avoid human error in determining degree of ossification, a single gray value, determined at a thin section of the parapophysitic sheet of the complex vertebra, was used for all elements in most scans. A small number of scans required separate gray values for thin and thick bone because of scan artifacts. All ossicles, the Weberian vertebrae, and unidentified nodules of density equivalent to bone in the vicinity of the Weberian apparatus were segmented, then exported as STL files.

### Landmark placement and principal component analysis

We created three landmark sets for the left scaphium (11 landmarks), left tripus (10 landmarks), and peripheral structures particularly the pars sustentaculum and attachment points of the inter-ossicular ligaments (25 landmarks) (Fig. 1). The landmark scheme was modified from Hoeflich and Liu (2025). Landmarking was performed by a single user (JH) to eliminate inter-personal variation, and afterward reassessed for consistent placement. Two landmarks, the most posterior point on the tripus and the dorsal margin of the posterior process of the scaphium, were excluded from the analyses because of low confidence in precision. Additionally, we removed specimens from the datasets if pathologies, highly unusual ossification patterns, or scan artifacts made the placement of all landmarks in a dataset impossible or highly inconsistent. As such, the scaphium dataset consisted of 137 individuals, the tripus dataset 134 individuals, and the peripheral dataset 127 individuals. The specimens that were excluded and the explanation for their exclusion is listed in Specimen Data Supplement. We placed the landmarks on the meshes in 3D Slicer (Fedorov et al. 2012), then exported the landmarks as mrk.jsn files and imported them into the GPA module of the Slicermorph extension, which created analysis.txt files for easy import into R. The mrk.json files are included in the supplemental materials.

**Fig. 1:**
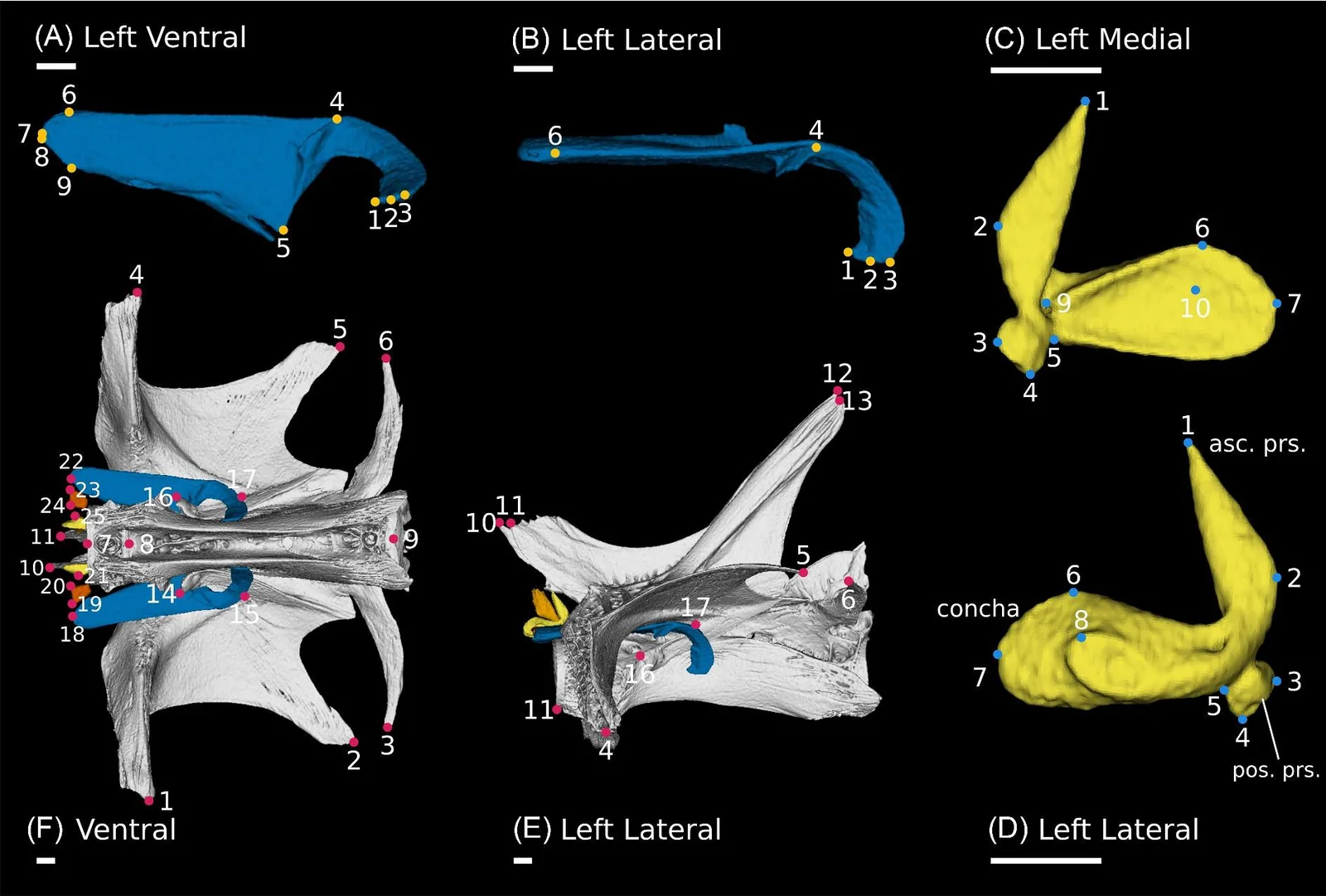
Placement of landmarks on the scaphium, tripus, and overall shape of peripheral structures. Landmarks sets are demonstrated on a specimen of *Ameiurus nebulosus* (ANSP 132296). (**A**) **and** (**B**) Tripus: 1, posterior process anterior inflection; 2, posterior process midway tip; 3, posterior process posterior inflection; 4, lateral tunica line; 5, medial posterior extent of anterior process; 6, lateral inflection of anterior process; 7, most anterior point; 8, anterior ligament 3 insertion; 9, posterior ligament 3 insertion. (**C**) **and** (**D**) Scaphium: 1, anterior tip ascending process; 2, widest point ascending process; 3, posterior tip posterior process; 4, ventral tip posterior process; 5, anterior edge posterior process; 6, highest point concha; 7, anteriormost point concha; 8, widest point concha; 9, lowest margin crux; 10, depth at widest point. (**E**) **and** (**F**) complex vertebra with ossicles: 1, right anterior parapophysis tip; 2, right middle parapophysis tip; 3, right posterior parapophysis tip; 4, left anterior parapophysis tip; 5, left middle parapophysis tip; 6, left posterior parapophysis tip; 7, centrum of first vertebra; 8, centrum of second vertebra; 9, centrum of last fused vertebra; 10, right anterior neural spine tip; 11, left anterior neural spine tip; 12, right posterior neural spine tip; 13, left posterior neural spine tip; 14, right distal anterior tip of os suspensorium; 15, left distal anterior tip of os suspensorium; 16, right proximal angle of os suspensorium; 17, left proximal angle of os suspensorium; 18, right medial anteriormost point of the tripus; 19, right anteriormost lateral point on intercalarium; 20, right anteriormost medial point on intercalarium; 21, right anterior insertion point on the scaphium; 22, left medial anteriormost point of the tripus; 23, left anteriormost lateral point on intercalarium; 24, left anteriormost medial point on intercalarium; 25, left anterior insertion point on the scaphium. The tripus and vertebral portion of the Weberian apparatus are presented in ventral (A, F) and lateral view (B, E), with the anterior of the fish to the left. The scaphium is presented in lateral view (D), with anterior to the left and dorsal up, as well as in medial view (C), with anterior to the right and dorsal up. The scale bars equal 1 mm.

All analyses were performed in R using the *geomorph* package (Adams and Otárola-Castillo 2013) unless otherwise noted. The methods for generating principal components (PCs) followed Hoeflich and Liu (2025), in which species-mean dataset was generated by averaging the Procrustes-aligned landmarks of multiple specimens for each species and then performing the calculation for PCs. As in the previous paper, all analyses accounted for phylogeny using the time-calibrated cladogram of Janzen et al. (2023), trimmed and collapsed to singular nodes for each of the species sampled. The phylogenetic position of recently extinct *N. trautmani* was not available (Janzen et al. 2023), and as such the species was excluded from all phylogenetic comparative analyses. All scripts for the geometric morphometrics and following analyses are included in the supplemental materials.

### Ecological association

We used the phylogenetic generalized least squares analyses with Analysis of Variance (ANOVA) for the full and species-mean datasets following those of Hoeflich and Liu (2025). The ecological correlation analysis examined four factors, the two which were found to be significant in Hoeflich and Liu (2025): stream velocity and dorsal coloration (as a proxy for substrate), and two new factors: tail morphology [as a proxy for lifestyle) and climate. *Prietella* was removed from the coloration analysis given the absence of pigmentation and the improbability of visual crypsis in absolute darkness. Dorsal coloration was divided into three categories as in Hoeflich and Liu (2025)]: solid, patched, and mottled. Tail morphology was assessed visually and sorted into two categories: forked, and truncate/rounded. Truncate and rounded morphologies were considered in the same category because of a lack of clear delineation between the two in ictalurid catfishes and intraspecific variation. Climate zones were defined by comparing recently compiled range maps (Varela-Romero et al. 2021; U.S. Geological Survey, 2026; IUCN, 2026), with Köppen–Geiger climate maps (Kottek et al. 2006). Climate was defined by the main climate throughout the species’ ranges: equatorial (A), arid (B), or warm temperate (C). No species showed a dominantly boreal (D) distribution, but several species possess ranges that extended well into that climate zone. As such, we also included warm temperate with boreal (C + D) as a category. Species that did not appear limited by climate zone (i.e., possessed ranges that significantly overlapped three or more climates) were excluded from this analysis. Similarly, if an ecological factor was unknown for a species, the species was removed from that particular analysis. In addition to these ecological factors, we also ran co-variation analyses using the genera and centroid sizes. *Pylodictis* and *Prietella* were excluded from the genera-grouped analysis because they were represented by a single species in this study. The classifier files for the assignment of ecological factors for each specimen and species are given in the supplemental materials.

### Phylogenetic independence and evolutionary rates

Phylogenetic independence was assessed using Blomberg’s *κ * (Blomberg et al. 2003) and calculated using the physignal function in the *geomorph* package for R (Adams and Otárola-Castillo 2013; Adams 2014). The analyses were performed three times for each landmark set with (1) all taxa shown in the phylogenetic tree (Janzen et al. 2023), (2) both single sampled species genera excluded, and (3) only *Pr. phreatophilia* excluded because of variable placement across phylogenies (Arce-H. et al. 2017; Janzen et al. 2023). Additionally, we tested for differences in rates of evolution among genera (*Ameiurus, Ictalurus*, and *Noturus*) using the compare.evol.rates function in *geomorph*.

We calculated the evolutionary rates along the branches of the phylogenetic tree using the *BayesTraits* V4.0.0 package (Meade and Pagel 2022). We employed a continuous Markov chain Monte Carlo variable rates model, largely following a similar protocol described by Evans et al. (2021). We used the first 11 PCs from the species-mean datasets, which accounted for over 96% of the total variation for all landmark sets. We ran three chains for each of the PC sets. Each chain ran for 10 million iterations and used the TestCorrel function to set the covariance between the PCs to zero. After running each chain, we assessed the data in Tracer v1.7.2 to determine the burn-in threshold (Rambaut et al. 2018). We used BayesTrees (Chipman et al. 2010) to discard the first 10% of trees as burn in and generate a consensus tree from the remainder of each chain. We then visually compared each of the three consensus trees from each PC set by mapping the rates onto the time-calibrated phylogeny using the *bnpsd* (Ochoa and Storey 2021) and *phytools* (Revell 2024) packages in R. The files associated with these analyses are included in the supplemental materials.

We also calculated disparity through time and compared it with simulated evolution along the phylogeny under Brownian motion model using the dtt function in *geiger*. The Brownian motion model was simulated 1000 times. For each landmark set, we ran the same analysis for all sample species of the family, then the genera, respectively.

### Morphological modularity

We tested for variational and evolutionary modularity (Zelditch and Goswami 2021) with each of the landmark sets (scaphium, tripus, and peripheral structures) representing a module. Specimens that did not have landmarks confidently placed for all three landmark sets were removed from this analysis. The vetted landmark dataset was Procrustes-aligned with species-mean landmark configurations calculated for each set, then combined into a single landmark set. In *geomorph*, we used the modularity.test and phylo.modularity to calculate the degree of modularity (CR coefficient) and multi.evol.rates functions to compare the rate of change along the phylogeny for each of the module (Adams and Collyer 2016). We did not test separately for integration using the partial least squares (PLS) test, because of the weakness of the test to small sample sizes and high number of variables. Both our sample size and large number of variables were well within the range shown to produce a high PLS score in a randomly-generated dataset (Adams and Felice 2014).

## Results

### Anatomical variations

The first three PCs for the species-mean datasets consistently covered more of the total variation than the full specimen data for the scaphium (64.5% vs. 56.0%), tripus (64.6% vs. 55.6%), and peripheral structures (75.8% vs. 70.3%) datasets, likely because intraspecific variation was obscuring some interspecific variation. Species of each genus occupy a distinctive area of the morphospace created by the first three PCs (Fig. 2). These trends are maintained in the analyses excluding *Ictalurus pricei* (Table S7). Generic affinity was highly significant and accounted for more than a third of the overall variation for the peripheral structures but only a small percentage for the scaphium and tripus (Fig. 2, Tables S1 and S6).

**Fig. 2:**
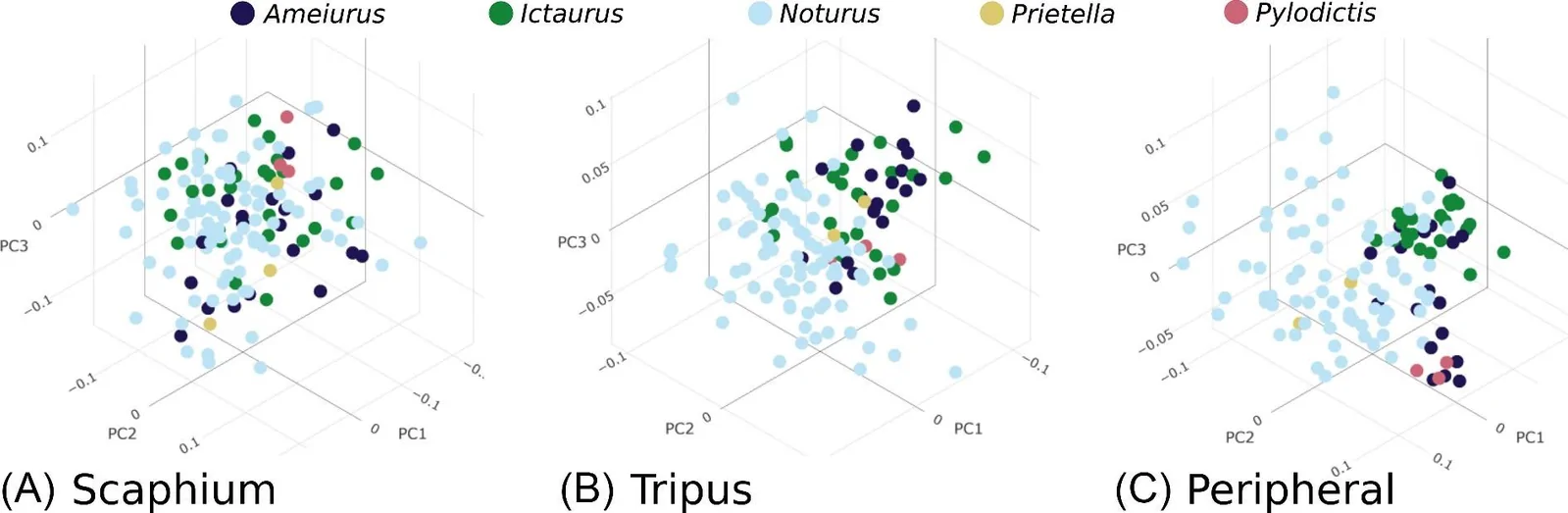
Distribution of genera in the morphospace created by first three PCs of (**A**) scaphium, (**B**) tripus, and (**C**) peripheral structures. The PC plots were generated by the full specimen dataset of each landmark scheme. The first three PC’s of the scaphium (**A**) dataset summarize 64.5% of the total variation (PC1: 28.4%, PC2: 16.0%, PC3: 11.5%), the tripus (**B**) summarize 64.6% (PC1: 25.1%, PC2: 17.2%, PC3: 13.3%), and the peripheral structures (**C**) summarize 75.8% (PC1: 42.9%, PC2: 20.6%, PC3: 6.8%).

The morphologies of the scaphium among ictalurids showed regional changes in the concha scaphii, ascending process, and posterior process. PC1 (28.5% of total variation in full specimen dataset, 34.7% of variation in species-mean dataset) of the scaphium describes the general robustness of the structure, with scaphia possessing thicker-walled, more elongate conchae, and more massive posterior processes on the negative end of the axis. Increasingly positive values along the PC2 axis (16.0% full, 18.3% species-mean) corresponded to more concave and overall larger concha. PC3 (11.5% full, 11.9% species-mean) described the size of the posterior process and the degree to which the tip of the ascending process was angled anteriorly, with more positive values indicating a larger posterior process and a forward-angled ascending process.

The major variation in the form of the tripus were shifts in the overall shape, rather than regional changes in the anterior or posterior processes. PC1 (25.1% full, 30.3% species-mean) describes an axis of compression, with positive values corresponding to dorso-ventral compression and negative values to lateral compression. Shifts along PC2 (17.2% full, 19.3% species-mean) produces diagonal transformations, with the lateral margin shifting posteriorly (positive) or anteriorly (negative) relative to those medial margin changes along the positive axis. Shifts in the length and robustness of the posterior process and the relative lengths of the anterior versus posterior process fell along PC3 (13.3% full, 15.0% species-mean), with positive values corresponding to shorter, less robust posterior processes.

The variation in the peripheral structures was dominated by changes in the form of the complex vertebra, particularly the parapophytic sheet and neural processes. Both PC1 and PC2 cover changes in the dorso-ventral compression of the complex vertebra. PC1 (43.0% full, 48.3% species-mean) describes co-occurring changes in the height of the posterior neural process and the ventral extent of the anterior parapophysis, with more positive values representing decreases in length of both processes. The overall dorso-ventral compression and antero-posterior length of the complex vertebra is summarized by PC2 (20.6% full, 20.6% species-mean), with longer, more compressed specimens in the positive. The positive changes along PC3 (6.7% full, 6.9% species-mean) warps the complex vertebra along a diagonal vector, with the parapophytic sheet shifting backward while the neural process extends forward.

### Impactful ecological factors

All three landmark sets produced mosaic patterns of correlation with the four tested ecological factors. All four factors are significantly correlated with the raw morphology, with each species represented by multiple specimens. However, the statistical significance (*P*-value) of the correlations between morphology and ecological factors fluctuated both during the compression of the dataset from individual specimens to species-mean values and during phylogenetic correction. (Table 1, Fig. S1, Table S8).

**Table 1:** Correlations between the morphology of Weberian ossicles and peripheral structures and ecological factors in Ictaluridae

	Scaphium			Tripus			Peripheral structures		
	Standard	Species-mean without phylogenetic framework	Species-mean with phylogenetic framework	Standard	Species-mean without phylogenetic framework	Species-mean with phylogenetic framework	Standard	Species-mean without phylogenetic framework	Species-mean with phylogenetic framework
Color pattern	0.002 ** *R*^2^ = 0.0407	0.085	0.001 ** *R*^2^ = 0.202	0.001 ** *R*^2^ = 0.0747	0.009 * *R*^2^ = 0.101	0.001 ** *R*^2^ = 0.196	0.001 ** *R*^2^ = 0.134	0.003 ** *R*^2^ = 0.148	0.013 * *R*^2^ = 0.117
Tail morphology	0.001 ** *R*^2^ = 0.0410	0.005 ** *R*^2^ = 0.0702	0.999	0.001 ** *R*^2^ = 0.0779	0.001 ** *R*^2^ = 0.101	0.998	0.001 ** *R*^2^ = 0.288	0.001 ** *R*^2^ = 0.331	0.35
Climate	0.003 ** *R*^2^ = 0.0526	0.159	0.011 * *R*^2^ = 0.205	0.001 ** *R*^2^ = 0.0591	0.359	0.002 ** *R*^2^ = 0.262	0.001 ** *R*^2^ = 0.175	0.005 ** *R*^2^ = 0.183	0.007 ** *R*^2^ = 0.173
Velocity	0.014 * *R*^2^ = 0.0202	0.14	0.001 ** *R*^2^ = 0.181	0.002 ** *R*^2^ = 0.0268	0.145	0.008 ** *R*^2^ = 0.1105	0.006 ** *R*^2^ = 0.0335	0.24	0.003 ** *R*^2^ = 0.108

Tail morphology was significant for all phylogenetically uncorrected analyses, but no longer significant when phylogeny is considered. In contrast, all other ecological factors are significant in association with all three tested structures when phylogeny is accounted for. Dorsal color pattern, reflecting substrate preference, stands out because it is robust in all three sub-tests for tripus and peripheral structures, whereas climate factors is significant across all tests for peripheral structures.

In all four series of testing, the proportion of variation accounted for by the variable (${R}^2$) increased once phylogeny was taken into account. This indicates these variables disproportionally account for deviations from what would be expected from phylogeny.

### Evolutionary tempo

All landmark datasets exhibited a statistically significant phylogenetic signal. However, the calculated ${K}_{\textit{mult}}$ values across all sets were substantially lower than 1.0 (full dataset: scaphium 0.21, *P* = 0.008; tripus, 0.22, *P* = 0.006; peripheral, 0.37, *P* = 0.001). These results indicate a weak phylogenetic structure, suggesting that phenotypic divergence is considerably greater than what would be expected under a Brownian motion model of evolution. (Tables S2 and S8).

The three datasets showed similar shifts in evolutionary rate (Fig. 3). There is an inverse relationship between divergence time and evolutionary rate in speciose genera including *Noturus* and *Ameiurus*, where many short branches show slow rates. Species, shown as terminal branches, in general display similar levels of evolutionary rates. The overall evolutionary rate of each speciose genus (*Ameiurus, Ictalurus*, and *Noturus*) are significantly different from each other in two out of three datasets (Tables S3 and S10). In the full dataset, the overall evolutionary rate of the scaphium is significantly different from both the tripus and peripheral structures, whereas the latter two do not significantly differ from each other (Tables S5 and S12).

**Fig. 3:**
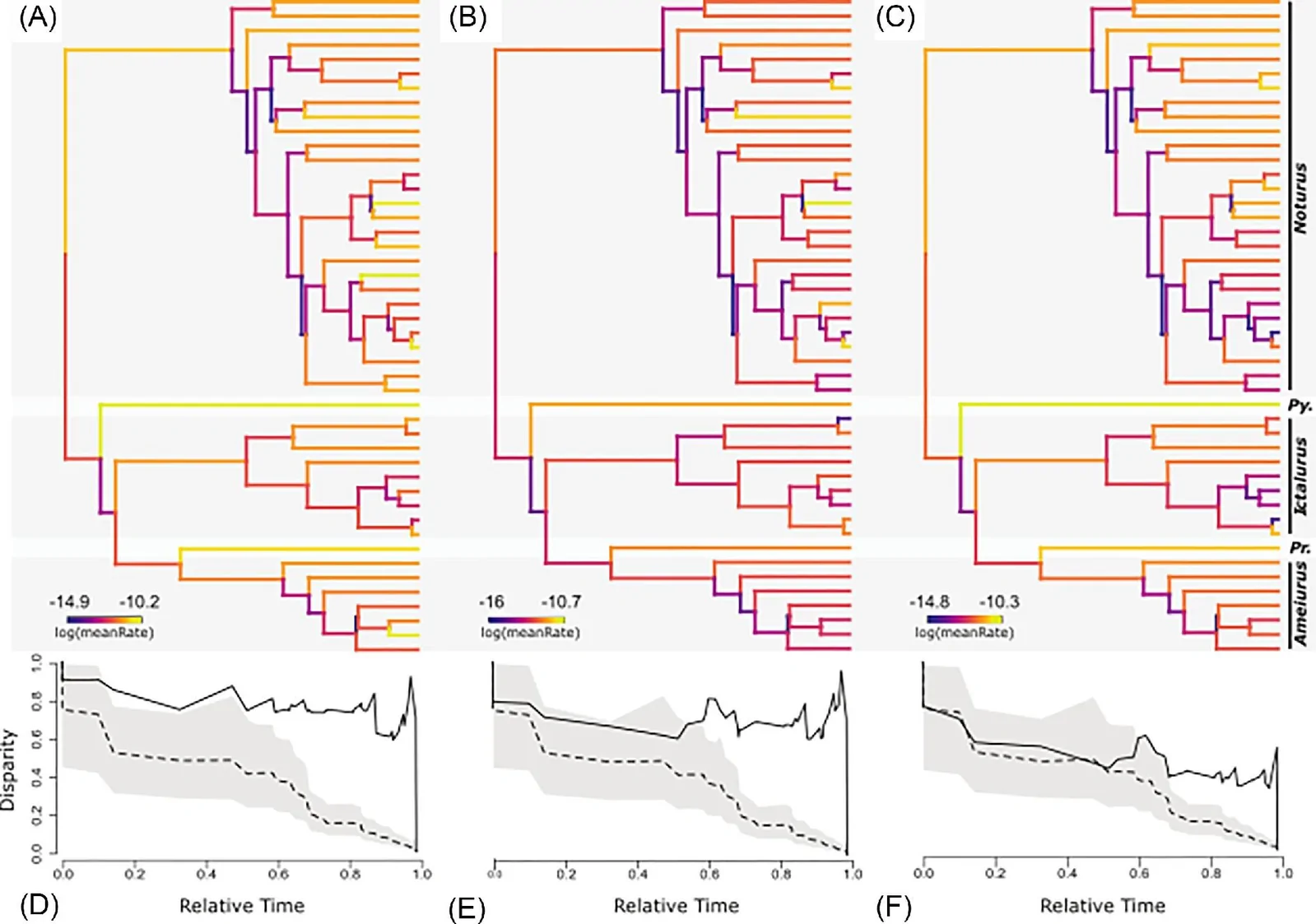
Evolutionary rate and morphological disparity across the ictalurid phylogeny. Evolutionary (**A, B**, and **C**) and average disparity through time (**D, E**, and **F**) for the scaphium (**A** and **D**), tripus (**B** and **E**), and peripheral structures (**C** and **F**).

The scaphium consistently showed higher morphological disparity than expected under a Brownian motion model, exceeding the disparity predicted under Brownian Motion early in the phylogeny (Fig. 3D). However, the morphological disparity of both the tripus and peripheral structures stayed with that predicted until relatively late in the phylogeny (Fig. 3E, F). The timing of these departures from Brownian Motion expectation for the tripus and peripheral structures coincide with the onset of rapid evolution of terminal branches of the Ictaluridae family. At the genus level, all datasets of *Ictalurus* and *Noturus* show similar patterns of tripus and peripheral structure disparity as at the family level. The disparity of all structures in *Ameiurus* is more or less within the prediction of Brownian Motion model (Fig. S3).

## Discussion

The Weberian apparatus is a key and transformative anatomical innovation in Otophysi. Seminal studies have been devoted to morphological diversification (Alexander 1962; Alexander 1964; Bird and Hernandez 2007; Boyle and Herrel 2018), deep time evolution (Chardon and Vandewalle 1996; Liu et al. 2025), hearing physiology (Weiss et al. 1969; Wysocki et al. 2009; Maiditsch and Ladich 2014; Putland et al. 2019), hearing mechanism (Weber 1819; Poggendorf 1952; Alexander 1962; Alexander 1964; Finneran et al. 2000; Marcé-Nogué and Liu 2024), and hearing ecology (Lechner and Ladich 2008; Mélotte et al. 2018; Hoeflich and Liu 2025). Our study is the first to integrate quantified morphology, ecological association, and evolutionary modeling in three discrete osteological components of the Weberian apparatus. Using Ictaluridae as a model system, our series of analyses present an integrative approach to tackle the complex eco-evo-morphology of the Weberian apparatus.

Ecological factors appear to be significant to morphological variation in the Weberian apparatus as evidenced by our findings that stream velocity, substrate (proxied by coloration), and climate correlate significantly with its morphology. This aligns with our general understandings that the energy of an aquatic environment shapes its soundscape and, consequently, the auditory sensitivity of resident fish (Amoser and Ladich 2005; Fay 2009). While morphological divergence in high- vs. low-velocity systems has been documented in serrasalmids (Boyle and Herrel 2018) and the ictalurid genus *Noturus* (Hoeflich and Liu 2025), alongside convergence in rheophilic Cypriniformes (Bird and Hernandez 2007), our study expands these observations across the family Ictaluridae. Notably, our results reaffirm that the correlation between substrate and Weberian ossicle morphology, first identified in *Noturus* (Hoeflich and Liu 2025), is a widespread familial trait rather than a generic peculiarity. Furthermore, we report a novel correlation between climate classification and Weberian morphology, a relationship previously untested in otophysans. This may reflect a direct climatic influence on the soundscape via water temperature (Putland et al. 2019), which could alter auditory sensitivity in *I. punctatus* (Wysocki et al. 2009) and other catfishes (Papes and Ladich 2011; Maiditsch and Ladich 2014; Schliwa and Ladich 2021), though it is equally possible that climate co-varies with other influential factors like invertebrate assemblages (Boix and Batzer 2016) or aquatic vegetation (Lacoul and Freedman 2011). This suggests that the Weberian apparatus may be even more plastic or evolutionarily responsive to complex ecosystem dynamics than previously assumed, and untangling these multifaceted interactions will require more granular climatological and ecological data.

The significance of ecological factors discussed above corroborate our phylogenetic structure tests, which revealed a significant but weak phylogenetic signal across all datasets; specifically, the uniformly low ${K}_{\textit{mult}}$ values indicate greater phenotypic divergence than would be expected under a Brownian Motion model (Blomberg et al. 2003). This aligns with the high average morphological disparity through time that generally exceeded Brownian Motion predictions across all three datasets (Fig. 2D–F). Consequently, these patterns suggest that evolutionary rates may vary among the three regions of the Weberian apparatus. Indeed, the results reveal significant differences in evolutionary rates across all regions, with the scaphium significantly outpacing the other modules, including the tripus. However, interpretation of these rates requires caution, particularly for ancient ancestral nodes, as our results indicate the apparatus is likely under ecological selective pressure, thereby violating the assumption of trait neutrality and introducing potential errors (Holland et al. 2020). Furthermore, because ancestral character states were calculated solely from extant data, they likely underestimate true historical morphological variation (Monson et al. 2022). Given that Ictaluridae possesses a robust fossil record (Lundberg 1975; Grande and Lundberg 1988; Grande 2001), future work would benefit from the incorporation of paleontological data to refine these evolutionary trajectories.

Evolutionary rate analyses have also revealed significant differences across genera, likely driven by variations in life history and geography. For example, many *Noturus* species exhibit high evolutionary rates potentially linked to their short generation times (often less than 2 years) and small endemic ranges, which increase susceptibility to stochastic events like bottlenecks (Taylor 1969; Burr and Warren Melvin L. 2020). However, these factors do not fully explain the observed variation; *Noturus* shows accelerated evolution only in the scaphium, and distinct rate differences persist between *Ameiurus* and *Ictalurus*. Disparity-through-time analyses further distinguish these groups; unlike *Ictalurus* and *Noturus*, which deviate significantly from Brownian Motion probability space, the traces for *Ameiurus* remain largely within or near the null space (Fig. S3). These results suggest that while other genera experience more variable evolutionary shifts, *Ameiurus* may be uniquely governed by neutral or stabilizing selection.

Our study further identified modularity across the three tested structures in all species (Fig. S4), which aligns with the significant differences in evolutionary rates observed between the defined modules (Fig. S5). Recent intraspecific research on the Weberian apparatus of *Ictalurus punctatus* also detected modularity, with the authors suggesting that this organization may have facilitated environmental adaptation (Duclos and Grande 2025). Similar investigations have been conducted on the Weberian apparatus of Cypriniformes. While studies have observed developmental (Bird, Richardson et al. 2020) and variational (Bird, Abels et al. 2020) integration, there is also evidence supporting modularity hypotheses (Duclos et al. 2021). No equivalent quantitative study has been performed in Cypriniformes, but most Cypriniformes lineages possess ossicular chains qualitatively similar to the generalized form of *Cyprinus carpio*. Some lineages do display more derived ossicular morphologies, with the highly reduced tripus of *Barbatula* representing a notable extreme (Alexander 1962; Bird and Hernandez 2007). Catfishes have been observed to show more extreme gross variation, with the loss of both processes and entire ossicles not uncommon (Lechner and Ladich 2008; Hoeflich and Liu 2025). This suggests that the mechanisms of variation, and consequently variational modularity may differ between Cypriniformes and Siluriformes. Studies of catfishes (Black and Armbruster 2022; Duclos and Grande 2025) and other ray-finned fishes (Larouche et al. 2018) have identified anatomical modularity as a primary element in diversification. For instance, functional modularity is evident in wrasse skulls (Larouche et al. 2023), though integration drives diversification in asymmetric flatfish (Evans et al. 2021). Within this context, the modularity we observed between the scaphium and tripus is surprising, as both ossicles share the same primary role: sound conduction (Chardon and Vandewalle 1996). Because integration usually accompanies shared function while modularity defines individualized units (Eble 2005; Zelditch and Goswami 2021), our findings imply that the scaphium and tripus may possess distinct functional roles or respond independently to different ecological selective pressures.

Synthesizing these results within a framework of evolvability indicates that ictalurids likely avoided the constraints of niche competition and priority effects (Jablonski 2022) due to their status as the sole freshwater catfish lineage throughout most of their range. This high evolvability is perhaps enabled by the system’s modularity (Le Maître et al. 2020); while we did not assess ontogeny directly, the independent variation observed in the scaphium, tripus, and peripheral structures aligns with developmental hypotheses linked to increased degrees of freedom (Duclos and Grande 2025). This capacity for individual components of the Weberian apparatus to decouple likely facilitates fine-tuned adaptation, a conclusion reinforced by the significant correlations between morphology and ecological factors found in our study. Ultimately, while the Weberian apparatus represents a complex system with high evolvability, the mechanism allowing for such independence despite the functional linkage of the scaphium and tripus remains unclear. Future research may bridge this diversity of form to specific functions to fully illuminate the underlying adaptive landscape.

## Supplementary Material

obag061_Supplemental_Files

## Data Availability

All data needed to evaluate the conclusions in the paper are present in the paper and/or the Supplementary Materials. All CT scan data are available on MorphoSource in Project 000656686. Additional data related to this paper may be requested from the authors.

## References

[bib1] Adams DC . 2014. A generalized K statistic for estimating phylogenetic signal from shape and other high-dimensional multivariate data. Syst Biol 63:685–97. 10.1093/SYSBIO/SYU030 24789073

[bib2] Adams DC, Collyer ML. 2016. On the comparison of the strength of morphological integration across morphometric datasets. Evolution 70:2623–31. 10.1111/EVO.13045 27592864

[bib3] Adams DC, Felice RN. 2014. Assessing trait covariation and morphological integration on phylogenies using evolutionary covariance matrices. PLoS One 9:e94335. 10.1371/JOURNAL.PONE.0094335 PMC3984176 24728003

[bib4] Adams DC, Otárola-Castillo E. 2013. geomorph: an r package for the collection and analysis of geometric morphometric shape data. Methods Ecol Evol 4:393–9. 10.1111/2041-210X.12035

[bib5] Alexander RMN . 1964. The structure of the weberian apparatus in the siluri. Proc Zool Soc Lond 142:419–40. 10.1111/j.1469-7998.1964.tb04507.x

[bib6] Alexander R, Mc N. 1962. The structure of the weberian apparatus in the cyprini. Proc Zool Soc Lond 139:451–73. 10.1111/J.1469-7998.1962.TB01839.X

[bib7] Amoser S, Ladich F. 2005. Are hearing sensitivities of freshwater fish adapted to the ambient noise in their habitats? J Exp Biol 208:3533–42. 10.1242/JEB.01809 16155225

[bib8] Arce-H. M, Lundberg JG, O’Leary MA. 2017. Phylogeny of the North American catfish family Ictaluridae (Teleostei: siluriformes) combining morphology, genes and fossils. Cladistics 33:406–28. 10.1111/CLA.12175 34715724

[bib9] Avila-Treviño E, Fernando Cardoza-Martínez G, Alonzo-Rojo F, Pérez-Rodríguez R. 2025. *Ictalurusnazas* sp. nov., a new species of North American catfish (Siluriformes, Ictaluridae) from Mexico. Zookeys 1248:33–51. 10.3897/ZOOKEYS.1248.151641 PMC12340532 40809553

[bib10] Bird NC, Abels JR, Richardson SS. 2020. Histology and structural integration of the major morphologies of the cypriniform Weberian apparatus. J Morphol 281:273–93. 10.1002/jmor.21097 31886901

[bib11] Bird NC, Hernandez LP. 2007. Morphological variation in the Weberian apparatus of Cypriniformes. J Morphol 268:739–57. 10.1002/jmor.10550 17591731

[bib12] Bird NC, Richardson SS, Abels JR. 2020. Histological development and integration of the Zebrafish Weberian apparatus. Dev Dyn 249:998–1017. 10.1002/DVDY.172 32243643

[bib13] Black CR, Armbruster JW. 2022. Evolutionary integration and modularity in the diversity of the suckermouth armoured catfishes. R Soc Open Sci 9:1–17. 10.1098/RSOS.220713. PMC9682303 36425524

[bib14] Blomberg SP, Garland T, Ives AR. 2003. Testing for phylogenetic signal in comparative data: behavioral traits are more labile. Evolution 57:717–45. 10.1111/J.0014-3820.2003.TB00285.X 12778543

[bib15] Boix D, Batzer D. 2016. Invertebrate assemblages and their ecological controls across the world’s freshwater wetlands. In: Invertebrates in freshwater wetlands: an international perspective on their ecology. Springer, Cham, p. 601–39. 10.1007/978-3-319-24978-0_17.

[bib16] Boyle KS, Herrel A. 2018. Relative size variation of the otoliths, swim bladder, and Weberian apparatus structures in piranhas and pacus (Characiformes: Serrasalmidae) with different ecologies and its implications for the detection of sound stimuli. J Morphol 279:1849–71. 10.1002/JMOR.20908 30443931

[bib17] Burgin CJ, Colella JP, Kahn PL, Upham NS. 2018. How many species of mammals are there? J Mammal 99:1–14. 10.1093/JMAMMAL/GYX147

[bib18] Burr BM, Warren Melvin L Jr 2020. Ictaluridae: north American catfishes. In: Freshwater fishes of North America. Baltimore: Johns Hopkins University Press. p. 23–100.

[bib19] Chardon M, Vandewalle P. 1996. Evolutionary trends and possible origin of the Weberian apparatus. Neth J Zool 47:383–403. 10.1163/156854297X00076

[bib20] Chipman HA, George EI, McCulloch RE. 2010. BART: Bayesian additive regression trees. Ann Appl Stat 4:266–98. 10.1214/09-AOAS285

[bib21] Duclos KK, Grande TC. 2025. Siluriform Weberian apparatus: modularity and integration. In: Catfishes, a highly diversified group, volume 1: their outstanding biology, vol. 1. Boca Raton: CRC Press. p. 83–104.

[bib22] Duclos KK, Grande TC, Cloutier R. 2021. Modularity of the Weberian apparatus in the zebrafish using micro-CT technology and 3-D geometric morphometrics. In: Pradel Alan, Denton John S S, and Janvier Philippe, editors. Ancient fishes and their living relatives: a tribute to John G. Maisey. Verlag Dr. Friedrich Pfeil.

[bib23] Eble GJ . 2005. Morphological modularity and macroevolution: conceptual and empirical aspects. In: Rasskin-Gutman Diego and Callebaut Werner, editors. Modularity: understanding the development and evolution of natural complex systems. Cambridge: MIT Press.

[bib24] Evans KM, Larouche O, Watson SJ, Farina S, Habegger ML, and Friedman M. 2021. Integration drives rapid phenotypic evolution in flatfishes. Proc Natl Acad Sci USA 118:e2101330118. 10.1073/pnas.2101330118 PMC8106320 33931506

[bib25] Fay R . 2009. Soundscapes and the sense of hearing of fishes. Integrative Zoology, 4:26–32. 10.1111/j.1749-4877.2008.00132.x. 21392274

[bib26] Fedorov A, Beichel R, Kalpathy-Cramer J, Finet J, Pujol S, Bauer C, Jennings D, Fennessy F, Sonka M, Buatti J et al. 2012. 3D Slicer as an image computing platform for the Quantitative Imaging Network. Magn Reson Imaging 30:1323–41. 10.1016/J.MRI.2012.05.001 PMC3466397 22770690

[bib27] Finneran JJ,; Mardi CH, Hastings MC. 2000. A mathematical analysis of the peripheral auditory system mechanics in the goldfish (*Carassius auratus*). J Acoust Soc Am 108:1308–21. 10.1121/1.1286099 11008831

[bib28] Fricke R, Eschmeyer WN, van der Laan van der Laan R. 2026. Eschmeyer’s catalog of fishes: genera, species, references. http://researcharchive.calacademy.org/research/ichthyology/catalog/fishcatmain.asp. Electronic version accessed Nov., 3, 2026.

[bib29] U.S. Geological Survey . 2026. Nonindigenous Aquatic Species Database, Gainesville, FL. http://nas.er.usgs.gov, Nov. 3, 2026 accessed.

[bib30] Grande L . 2001. An updated review of the fish faunas from the green river formation, the world’s most productive freshwater lagerstätten. In: Eocene Biodiversity. Topics in Geobiology, vol 18. Springer, Boston, MA. p. 1–38. 10.1007/978-1-4615-1271-4_1.

[bib31] Grande L, Lundberg JG. 1988. Revision and redescription of the genus *Astephus* (Siluriformes: ictaluridae) with a discussion of its phylogenetic relationships. J Vertebr Paleontol 8:139–71. 10.1080/02724634.1988.10011694

[bib32] Hoeflich JC, Liu J. 2025. Small fish, large variation: morphological diversity of Weberian apparatus in *Noturus* catfishes and ecological implications. Anat Rec 308:3069–81. 10.1002/AR.25629 PMC12594186 39834136

[bib33] Holland BR, Ketelaar-Jones S, O’Mara AR, Woodhams MD, Jordan GJ. 2020. Accuracy of ancestral state reconstruction for non-neutral traits. Sci Rep 10:1–10. 10.1038/s41598-020-64647-4 PMC7203120 32376845

[bib34] IUCN . 2026. *The IUCN Red List of Threatened Species. Version 2026-1*. https://www.iucnredlist.org. Accessed on Nov. 3, 2026.

[bib35] Jablonski D . 2022. Evolvability and macroevolution: overview and synthesis. Evol Biol 49:265–91. 10.1007/S11692-022-09570-4

[bib36] Janzen FH, Pérez-Rodríguez R, Domínguez-Domínguez O, Hendrickson DA, Sabaj MH, Blouin-Demers G. 2023. Phylogenetic relationships of the North American catfishes (Ictaluridae, Siluriformes): investigating the origins and parallel evolution of the troglobitic species. Mol Phylogenet Evol 182:107746. 10.1016/J.YMPEV.2023.107746 36849094

[bib37] Kappas I, Vittas S, Pantzartzi CN, Drosopoulou E, Scouras ZG. 2016. A time-calibrated mitogenome phylogeny of catfish (Teleostei: Siluriformes). PLoS One 11:e0166988. 10.1371/JOURNAL.PONE.0166988 PMC5132296 27907107

[bib38] Kottek M, Grieser J, Beck C, Rudolf B, Rubel F. 2006. World Map of the Köppen–Geiger climate classification updated. Meteorol Z. 15:259–63. 10.1127/0941-2948/2006/0130

[bib39] Lacoul P, Freedman B. 2011. Environmental influences on aquatic plants in freshwater ecosystems. Environ Rev 14:89–136. 10.1139/A06-001

[bib40] Larouche O, Gartner SM, Westneat MW, Evans KM. 2023. Mosaic evolution of the skull in labrid fishes involves differences in both tempo and mode of morphological change. Syst Biol 72:419–32. 10.1093/SYSBIO/SYAC061 36111797

[bib41] Larouche O, Zelditch ML, Cloutier R. 2018. Modularity promotes morphological divergence in ray-finned fishes. Sci Rep 8:1–6. 10.1038/s41598-018-25715-y PMC5940925 29740131

[bib42] Lechner W, Ladich F. 2008. Size matters: diversity in swimbladders and Weberian ossicles affects hearing in catfishes. J Exp Biol 211:1681–9. 10.1242/jeb.016436 18456895

[bib43] Le Maître A, Grunstra NDS, Pfaff C, Mitteroecker P. 2020. Evolution of the mammalian ear: an evolvability hypothesis. Evol Biol 47:187–92. 10.1007/S11692-020-09502-0/METRICS PMC7399675 32801400

[bib44] Liu J, Brinkman DB, Murray AM et al. 2025. Marine origins and freshwater radiations of the otophysan fishes. Science 390:65–9. 10.1126/science.adr4494 41037602

[bib45] Lundberg JG . 1975. The Fossil Catfishes of North America. Claude W. Hibbard Memorial Volume II. University of Michigan, Ann Arbor. p. 1–60.

[bib46] Lundberg JG . 1982. The comparative anatomy of the toothless blindcat, *Trogloglanis pattersoni* Eigenmann, with a phylogenetic analysis of the ictalurid catfishes. Misc Public Univ Mich Mus Zool: 163. p. 1–85.

[bib47] Lundberg JG, Hendrickson DA, Luckenbill KR, Mariangeles AH. 2017. Satan’s skeleton revealed: a tomographic and comparative osteology of *Satan eurystomus*, the subterranean Widemouth Blindcat (Siluriformes, Ictaluridae). Proc Acad Nat Sci Philadelphia 165:117–73. 10.1635/053.165.0108

[bib48] Maiditsch IP, Ladich F. 2014. Effects of temperature on auditory sensitivity in eurythermal fishes: common carp *Cyprinus carpio* (Family Cyprinidae) versus Wels catfish *Silurus glanis* (Family Siluridae). PLoS One 9:e108583. 10.1371/JOURNAL.PONE.0108583 PMC4177911 25255456

[bib49] Marcé-Nogué J, Liu J. 2024. Finite element modelling of sound transmission in the Weberian apparatus of zebrafish (*Danio rerio*). J R Soc Interface. 21:20230553. 10.1098/RSIF.2023.0553. PMC10777150 38196376

[bib50] Meade A, Pagel M. 2022. Ancestral state reconstruction using BayesTraits. Methods Mol Biol 2569:255–66. 10.1007/978-1-0716-2691-7_12 36083452

[bib51] Mélotte G, Parmentier E, Michel C, Herrel A, Boyle K. 2018. Hearing capacities and morphology of the auditory system in Serrasalmidae (Teleostei: Otophysi). Sci Rep 8:1–11. 10.1038/s41598-018-19812-1 PMC5775314 29352233

[bib52] Monson TA, Brasil MF, Mahaney MC, Schmitt CA, Taylor CE, Hlusko LJ. 2022. Keeping 21st century paleontology grounded: quantitative genetic analyses and ancestral state reconstruction re-emphasize the essentiality of fossils. Biology 11:1218. 10.3390/BIOLOGY11081218/S1 PMC9404954 36009845

[bib53] Nelson JS, Grande T, Wilson MVH. 2016. Fishes of the world. 5th ed. Hoboken: John Wiley & Sons.

[bib54] Ochoa A, Storey JD. 2021. Estimating *FST* and kinship for arbitrary population structures. PLoS Genet 17:e1009241. 10.1371/journal.pgen.1009241 PMC7846127 33465078

[bib55] Papes S, Ladich F. 2011. Effects of temperature on sound production and auditory abilities in the striped raphael catfish *Platydoras armatulus* (Family Doradidae). PLoS One 6:e26479. 10.1371/JOURNAL.PONE.0026479 PMC3195728 22022618

[bib56] Poggendorf D . 1952. Die absoluten Horschwellen des Zwergwelses (*Amiurus nebulosus*) und Beitrage zur Physik des Weberschen Apparates der Ostariophysen. Z Vergl Physiol 34:222–57. 10.1007/BF00298202

[bib57] Putland RL, Montgomery JC, Radford CA. 2019. Ecology of fish hearing. J Fish Biol 95:39–52. 10.1111/JFB.13867 30447064

[bib58] Rambaut A, Drummond AJ, Xie D, Baele G, Suchard MA. 2018. Posterior summarization in Bayesian phylogenetics using Tracer 1.7. Syst Biol 67:901–4. 10.1093/sysbio/syy032 PMC6101584 29718447

[bib59] Revell LJ . 2024. phytools 2.0: an updated R ecosystem for phylogenetic comparative methods (and other things). PeerJ 12:e16505. 10.7717/PEERJ.16505 PMC10773453 38192598

[bib60] Schliwa M, Ladich F. 2021. Temperature (but not acclimation) affects hearing in fishes adapted to different temperature regimes. Comp Biochem Physiol A: Mol Integr Physiol 261:111053. 10.1016/J.CBPA.2021.111053 34375742

[bib61] Taylor WR . 1969. A revision of the catfish genus *Noturus* Rafinesque with an analysis of higher groups in the Ictaluridae William Ralph Taylor. United States. National Museum. Bulletin; 282. Smithsonian Institution Press.

[bib62] Teugels GG . 1996. Taxonomy, phylogeny and biogeography of catfishes (Ostariophysi, Siluroidei): an overview. Aquat Living Resour 9:9–34. 10.1051/ALR:1996039

[bib63] Thermo Scientific . 2021. Avizo 2021. Waltham, MA, USA.

[bib64] Varela-Romero A, Alonso Ballesteros-Córdova C, Ruiz-Campos G, Sánchez-González S, Brooks JE. 2021. Recent discoveries and conservation of catfishes, genus *Ictalurus*, in México. Standing between life and extinction. Chicago: University of Chicago Press. p. 285–294. 10.7208/CHICAGO/9780226694504.003.0019.

[bib65] Weber EH . 1819. Vergleichende Anatomie der Gehörwerkzeuge. Deutsches Archiv für Die Physiologie 5:323–32.

[bib66] Weiss BA, Strother WF, Hartig GM. 1969. Auditory sensitivity in the bullhead catfish (*ictalurus nebulosis*)*. Proc Natl Acad Sci USA 64:552–6. 10.1073/PNAS.64.2.552 PMC223379 5261032

[bib67] Wysocki LE, Montey K, Popper AN. 2009. The influence of ambient temperature and thermal acclimation on hearing in a eurythermal and a stenothermal otophysan fish. J Exp Biol 212:3091–9. 10.1242/JEB.033274 19749101

[bib68] Zelditch ML, Goswami A. 2021. What does modularity mean?. Evol Dev 23:377–403. 10.1111/EDE.12390 34464501

