# Supplementary material for: Soundscapes and Selection: Morphology, Ecology, and Evolutionary Dynamics of Weberian Apparatus in the Ictalurid Catfish
Source: Integr Org Biol. 2026 Oct 19;8(1):obag061. doi: 10.1093/iob/obag061 (PMC13769271; doi:10.1093/iob/obag061)
Supplement: Supplementary file 1 [file obag061_supplemental_files.zip › Ictaluridae_GMM_OUB_Supplement_Revision.pdf]

Extended Results

Table S1: Correlations between the morphology of Weberian ossicles and peripheral structures and genus identity in Ictaluridae.

	Standard	Species-Mean without Phylogenetic Framework	Species-Mean with Phylogenetic Framework
Scaphium	**0.001 ****<br>R^2 = 0.09566	**0.001 ****<br>R^2 = 0.17358	1
Tripus	**0.001 ****<br>R^2 = 0.16645	**0.001 ****<br>R^2 = 0.23064	1
Peripheral Structures	**0.001 ****<br>R^2 = 0.36507	**0.001 ****<br>R^2 = 0.42147	0.984

Table S2: Degree of phylogenetic structure in morphology. Tests for phylogenetic signal, κ_{mult}, for the scaphium, tripus, and peripheral structures, with analyses including all extant species, all excluding *Prietella*, and only genera for which multiple species were sampled.

	All (K, p)	Without *Prietella* (K, p)	Without *Prietella* or *Pylodictis* (K, p)
Scaphium	0.2121, **0.008***	0.2111, **0.006***	0.2055, **0.022***
Tripus	0.2155, **0.006***	0.2209, **0.01***	0.2191, **0.004***
Peripheral Structures	0.3676, **0.001***	0.3765, **0.001***	0.3679, **0.001***

Table S3: Rate of morphological evolution of the scaphium, tripus, and peripheral structures within *Ameiurus*, *Ictalurus*, *Noturus*.

	Evolutionary Rates			P-values		
	Ameiurus	*Ictalurus*	*Noturus*	*Ameiurus* vs. *Ictalurus*	*Ameiurus* vs. *Noturus*	*Ictalurus* vs. *Noturus*
Scaphium	2.70e-05	7.67e-05	5.86e-05	**0.001***	**0.001***	0.193
Tripus	5.83e-06	3.65e-05	2.81e-05	**0.001***	**0.001***	0.228
Peripheral Structures	7.73e-06	1.77e-05	9.98e-06	**0.002***	0.190	**0.004***

Table S4: Degree of modularity, measured with the co-variance ratio (CR), of the scaphium, tripus, and peripheral structures.

	p-value	Overall CR	Scaphium vs. Peripheral CR	Tripus vs. Peripheral CR	Scaphium vs. Tripus CR
Standard Modularity	**0.001 ***	0.7249	0.6057396	0.8305760	0.7384177
Phylogenetic Modularity	**0.001 ***	0.8142	0.7671703	0.8186518	0.8567798

Table S5: Rate of morphological evolution of the scaphium, tripus, and peripheral structures.

Evolutionary Rates			P-values		
Scaphium	Tripus	Peripheral	Scaphium vs. Peripheral	Tripus vs. Peripheral	Scaphium vs. Tripus
2.08e-05	8.78e-06	3.59e-06	**0.001 ***	0.378	**0.001 ***

Fig. S1: Distribution of ecological factors throughout the PC morphospaces. Principal components plots depicting the distribution of the full specimen dataset in the (a,d,g,j) scaphium (PC1: 28.4% var., PC2: 16.0% var., PC3: 11.5% var.), (b,e,h,k) tripus (PC1: 25.1% var., PC2: 17.2% var., PC3: 13.3% var.), (c,f,i,l) peripheral structures PC1 morphospaces (PC1: 42.9% var., PC2: 20.6% var., PC3: 6.8% var.), colored by color pattern (a, b, & c), tail morphology (d, e, & f), and climate (g, h, & i).

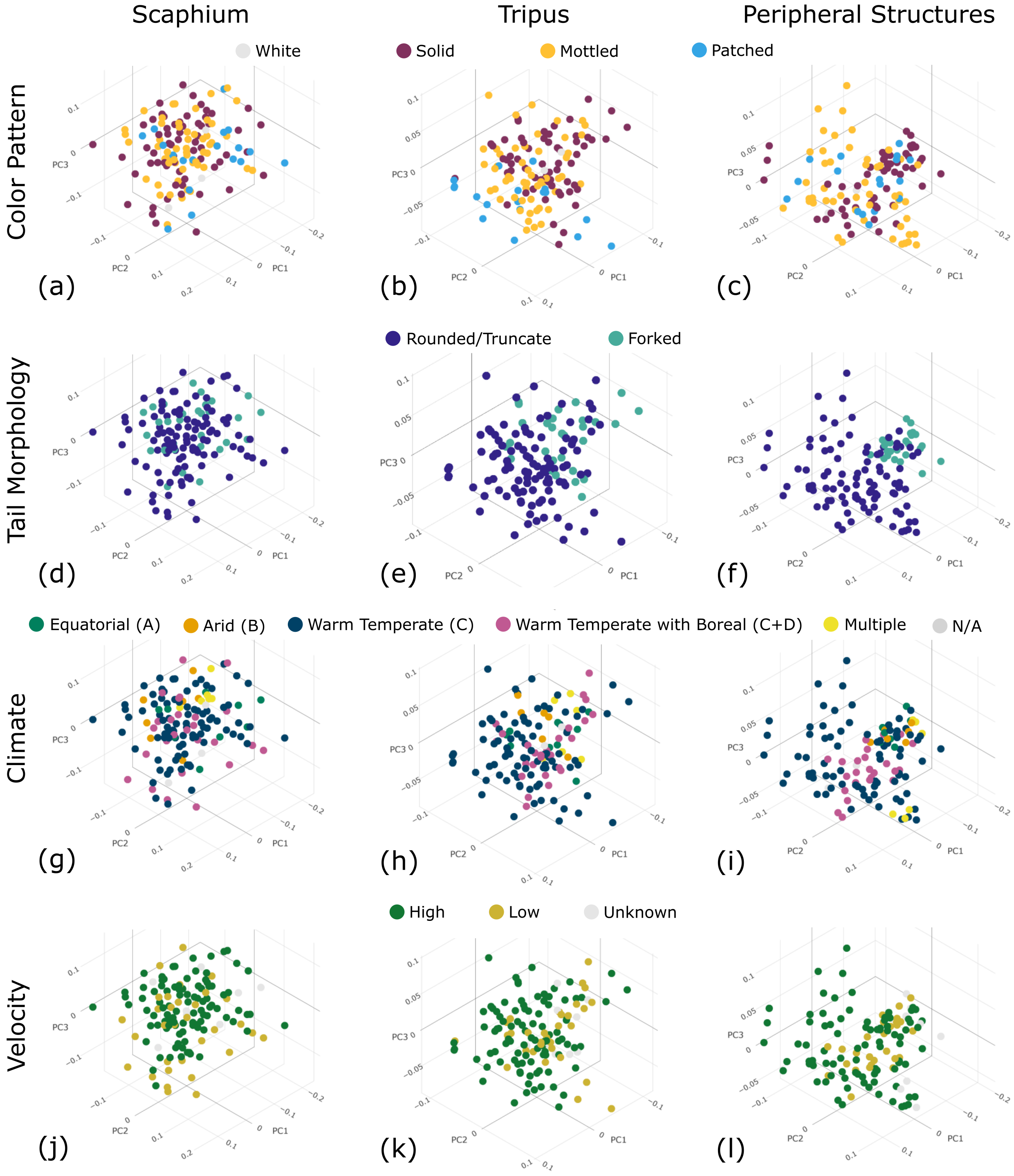

Fig. S2: Time to model convergence for variable-rates Bayestraits analyses. The iterative model likelihoods are given for the scaphium (yellow), tripus (pink), and peripheral structures (blue). The first 10% of each run was discarded as burn-in.

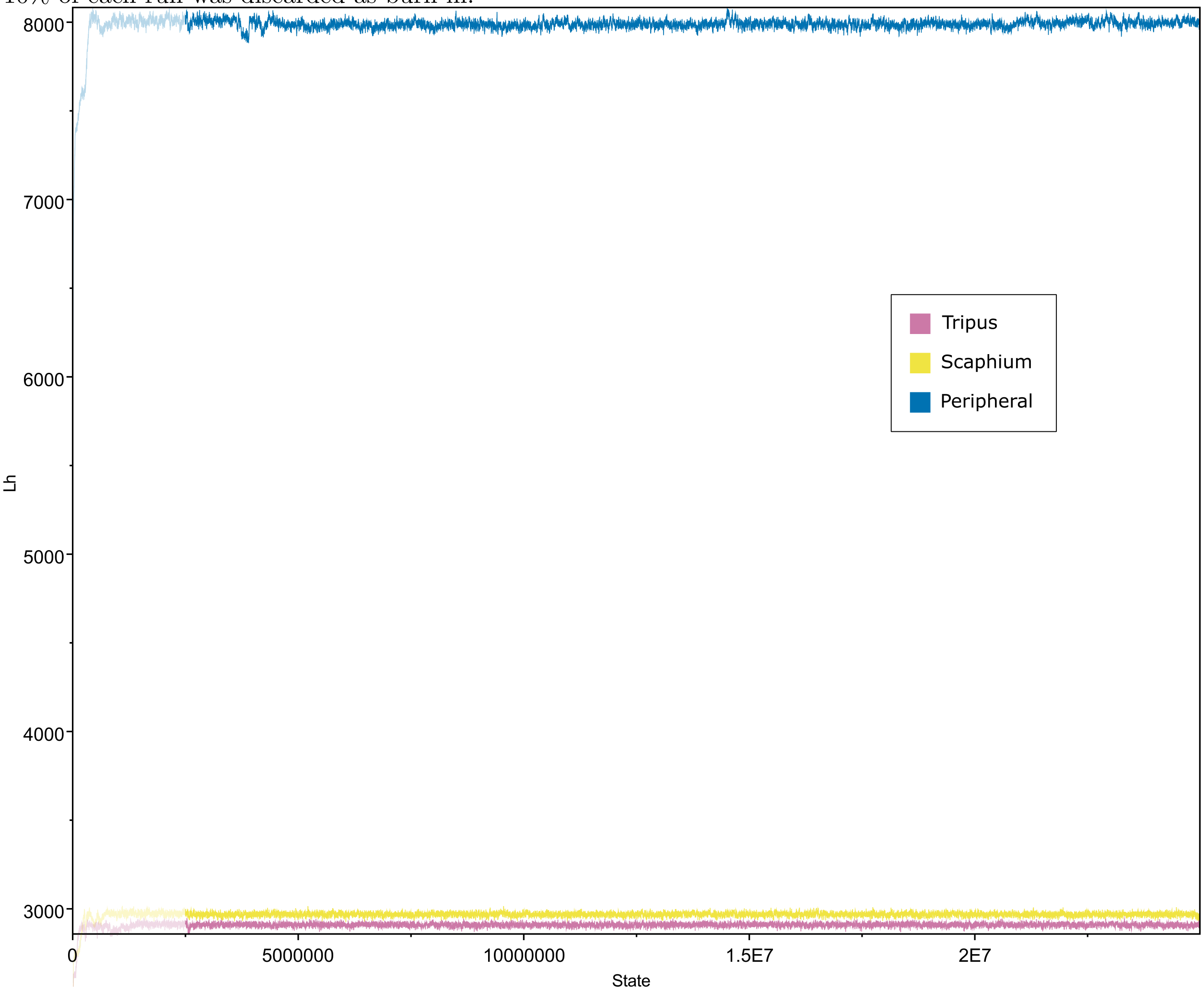

Fig. S3: Morphological disparity across time for ictalurid genera. Results from the *Ameiurus* (a,b, & c), *Ictalurus* (d, e, & f), and *Noturus* (g, h, & i) disparity through time analyses for the scaphium (a, d, & g), tripus (b, e, & h), and peripheral structures (c, f, & i).

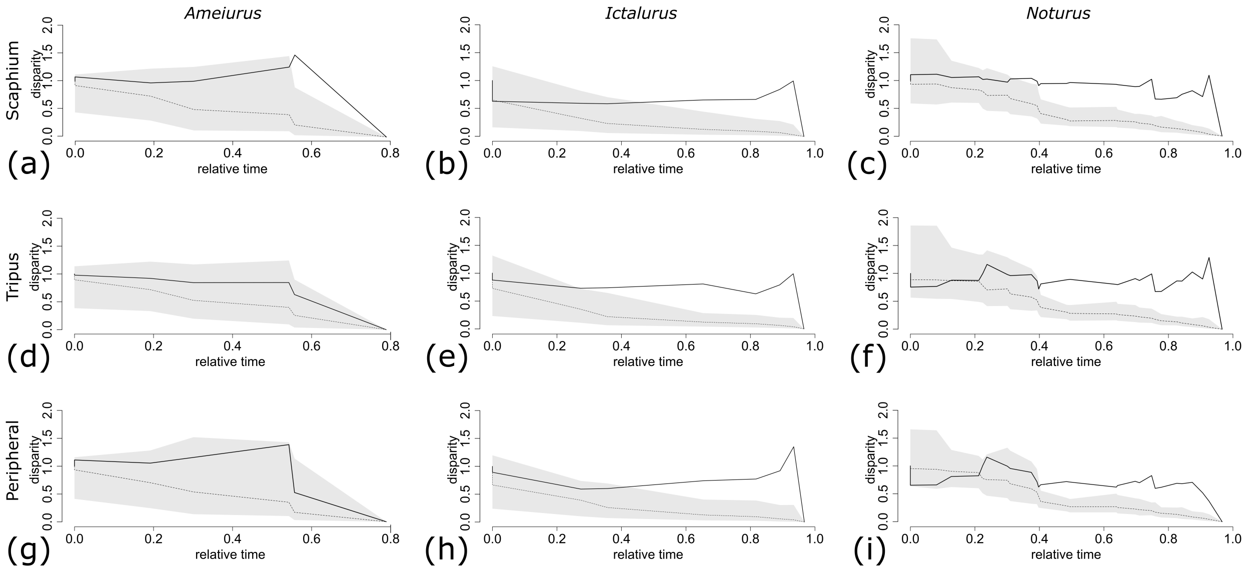

Results with *Ictalurus pricei* specimens removed

Table S6: Amount of morphological variation accounted for by the first three principal components for the scaphium, tripus, and peripheral structures after removal of *I. pricei* specimens.

		PC1	PC2	PC3	First Three
Scaphium	Standard	28.6%	15.8%	11.8%	56.2%
	Species-Mean	35.2%	17.7%	11.7%	64.6%
Tripus	Standard	25.1%	17.2%	13.3%	55.6%
	Species-Mean	30.1%	19.5%	15.1%	64.7%
Peripheral Structures	Standard	42.2%	20.9%	6.9%	70.0%
	Species-Mean	47.4%	21.0%	7.0%	75.4%

Table S7: Correlations between the morphology of Weberian ossicles and peripheral structures and genus identity in Ictaluridae after removal of *I. pricei* specimens.

	Standard	Species-Mean without Phylogenetic Framework	Species-Mean with Phylogenetic Framework
Scaphium	**0.001 **** R^2 = 0.09727	**0.001 **** R^2 = 0.17553	1
Tripus	**0.001 **** R^2 = 0.16447	**0.001 **** R^2 = 0.22797	1
Peripheral Structures	**0.001 **** R^2 = 0.35624	**0.001 **** R^2 = 0.41141	0.973

Table S8: Correlations between the morphology of Weberian ossicles and peripheral structures and ecological factors in Ictaluridae after removal of *I. pricei* specimens.

	Scaphium			Tripus			Peripheral Structures		
	Standard	Species-Mean without Phylogenetic Framework	Species-Mean with Phylogenetic Framework	Standard	Species-Mean without Phylogenetic Framework	Species-Mean with Phylogenetic Framework	Standard	Species-Mean without Phylogenetic Framework	Species-Mean with Phylogenetic Framework
Color Pattern	**0.01 **** R^2 = 0.0392	0.111	**0.001 **** R^2 = 0.205	**0.001 **** R^2 = 0.205	**0.001 **** R^2 = 0.205	**0.003 **** R^2 = 0.197	**0.001 **** R^2 = 0.125	**0.005 **** R^2 = 0.139	**0.01 *** R^2 = 0.119
Tail Morphology	**0.001 **** R^2 = 0.0405	**0.016 **** R^2 = 0.0692	0.999	**0.001 **** R^2 = 0.0750	**0.002 **** R^2 = 0.0974	0.997	**0.001 **** R^2 = 0.277	**0.001 **** R^2 = 0.318	0.38
Climate	**0.006 **** R^2 = 0.0473	0.293	**0.04 *** R^2 = 0.201	**0.001 **** R^2 = 0.0595	0.322	**0.003 **** R^2 = 0.275	**0.001 **** R^2 = 0.159	**0.011 **** R2 = 0.164	**0.038 **** R^2 = 0.158
Velocity	**0.016 *** R^2 = 0.0201	0.16	**0.001 **** R^2 = 0.181	**0.003 **** R^2 = 0.0280	0.122	**0.007 **** R^2 = 0.113	**0.004 **** R^2 = 0.0392	0.19	**0.004 **** R^2 = 0.108

Table S9: Degree of phylogenetic structure in morphology after removal of *I. pricei* specimens. Tests for phylogenetic signal, κ_{mult}, for the scaphium, tripus, and peripheral structures, with analyses including all extant species, all excluding *Prietella*, and only genera for which multiple species were sampled.

	All (K, p)	Without *Prietella* (K, p)	Without *Prietella* or *Pylodictis* (K, p)
Scaphium	0.212, **0.026***	0.2113, **0.021**	0.2054, **0.038***
Tripus	0.2153, **0.018***	0.2209, **0.015***	0.2192, **0.011***
Peripheral Structures	0.3655, **0.001***	0.3751, **0.001***	0.3686, **0.001***

Table S10: Rate of morphological evolution of the scaphium, tripus, and peripheral structures within *Ameiurus*, *Ictalurus*, *Noturus* after removal of *I. pricei* specimens

	Evolutionary Rates			P-values		
	Ameiurus	*Ictalurus*	*Noturus*	*Ameiurus* vs. *Ictalurus*	*Ameiurus* vs. *Noturus*	*Ictalurus* vs. *Noturus*
Scaphium	2.70e-05	8.40e-05	5.86e-05	**0.001***	**0.001***	0.093
Tripus	5.83e-06	4.09e-05	2.82e-05	**0.001***	**0.001***	0.108
Peripheral Structures	7.73e-06	1.97e-05	9.98e-06	**0.002***	0.180	**0.002***

Table S11: Degree of modularity, measured with the co-variance ratio (CR), of the scaphium, tripus, and peripheral structures after removal of *I. pricei* specimens.

	p-value	Overall CR	Scaphium vs. Peripheral CR	Tripus vs. Peripheral CR	Scaphium vs. Tripus CR
Standard Modularity	**0.001 ***	0.7291	0.6124637	0.8315094	0.7431973
Phylogenetic Modularity	**0.001 ***	0.8159	0.7696222	0.8213989	0.8565611

Table S12: Rate of morphological evolution of the scaphium, tripus, and peripheral structures after removal of *I. pricei* specimens.

Evolutionary Rates			P-values		
Scaphium	Tripus	Peripheral	Scaphium vs. Peripheral	Tripus vs. Peripheral	Scaphium vs. Tripus
2.11e-05	8.93e-06	3.62e-06	**0.001 ***	0.339	**0.001 ***

Supplemental Data

Data S1.
Specimen_Data_Supplement: CSV spreadsheet containing all specimen data collected for this study, with CT scan information and notes on usage in this study.

Data S2.
Landmark_Files: Zipped folder containing all landmarks used in this study, separated into subfolders by landmark set. The landmarks are saved in the mrk.json format.

Data S3.
Code_for_GMM_Supp: Code for all geometric morphometric analyses using individual landmark sets used in this paper, saved in the R format.

Data S4.
Evomodularity_Supp_Code: Code for geometric morphometric analyses related to modularity, saved in the R format.

Data S5.
Classifier_Ictaluridae_specimens: CSV spreadsheet using in analyses, containing ecological factor data according to species for each species in the species-mean analyses.

Data S6.
Classifier_Ictaluridae_specimens: CSV spreadsheet using in analyses, containing ecological factor data according to species for each specimen.

Data S7.
BayesTrees_Outputs: Zipped folder containing the output trees for the scaphium, tripus, and peripheral structures analyses from *Bayestrees* showing evolutionary rate, in newick format.
